# Labrune Syndrome: A Rare Leukodystrophy

**DOI:** 10.7759/cureus.39287

**Published:** 2023-05-21

**Authors:** Jishnu Nair, Sai Sriram Swamiyappan, Rav Tej Bathala, Jayesh Gupta, Kapil D Nayar, Balasubramaniam D

**Affiliations:** 1 Neurosurgery, Sri Ramachandra Institute of Higher Education and Research, Chennai, IND; 2 Neurosurgery, Sri Ramachandra Institute of Higher Education and Research, chennai, IND; 3 Internal Medicine, Sri Ramachandra Institute of Higher Education and Research, Chennai, IND

**Keywords:** snord118 gene mutation, labrune syndrome, cysts, cerebral calcifications, leukodystrophy

## Abstract

Labrune syndrome is a rare neurological disorder, with less than 100 reported cases since its identification. This disorder causes progressive cerebral degeneration. This case report describes a 21-year-old male patient who presented with tonic-clonic seizures. Upon examination, he was found to have symmetrical dense calcifications in the bilateral basal ganglia, thalami, and dentate nuclei, as well as in the white matter of both hemispheres, accompanied by cysts. MRI brain revealed confluent areas of T2/FLAIR hyperintensities involving the deep periventricular white matter in both cerebral hemispheres with sparing of subcortical U-fibres and two cysts in the left frontal and right posterior temporal region. No serologic evidence of a parasitic infection was found. Treatment was directed at addressing symptoms, and surgery was not required as the cysts were not causing a mass effect. The condition is the result of an autosomal mutation in the SNORD118 gene, a non-protein encoding gene that mediates rRNA synthesis.

## Introduction

First described by Labrune in 1996 in three pediatric patients, the disease had imaging features such as white matter degeneration with cysts along with calcification [1]. While pathologically, there is a microangiopathy characterized by angiomatous gliosis along with Rosenthal fiber deposition; the presentation is clinically of progressive cerebral degeneration. CT evaluation shows widespread calcifications in bilateral basal ganglia and bilateral deep cerebellar nuclei [1]. MRI reveals a significant amount of abnormal hyperintensity in the white matter, indicative of leukoencephalopathy [1]. When large parenchymal cysts form, they can put pressure on nearby structures and cause compression, which may necessitate surgical intervention [2,3]. Recent advancements in genetic studies have found an association with mutation in the SNORD118 gene [4]. Being a rare disease, theories regarding the etiology are still a matter of debate. Histopathological characteristics consist of obliterative microangiopathy [3]. Theories have postulated that obliterative microangiopathy leads to necrosis which in turn leads to the formation of cysts. The calcification seen is dystrophic [3,5]. Inherent alterations in the water content have been hypothesized to cause the changes seen in the white matter tracts rather than a primary pathology involving myelin [5]. We report a case of leukoencephalopathy with brain calcifications and cysts (LCC, also known as Labrune syndrome) and discuss the findings.

## Case presentation

A 21-year-old male with a normal developmental history presented with one episode of tonic-clonic movements of all four limbs lasting for a few minutes associated with the involuntary passage of urine and stools and post-ictal confusion. There was no history of antecedent trauma, and he had no focal neurological deficits. Visual field acuity and fundus examinations were unremarkable.

Complete blood count, erythrocyte sedimentation rate (ESR), calcium, liver and renal function tests, alkaline phosphatase, lactate, phosphate, serum thyroid, and parathyroid hormones were within reference ranges. Cytomegalovirus, hydatid cyst, Toxoplasma gondi, and HIV 1 and 2 serological tests were found to be negative.

**Figure 1:**
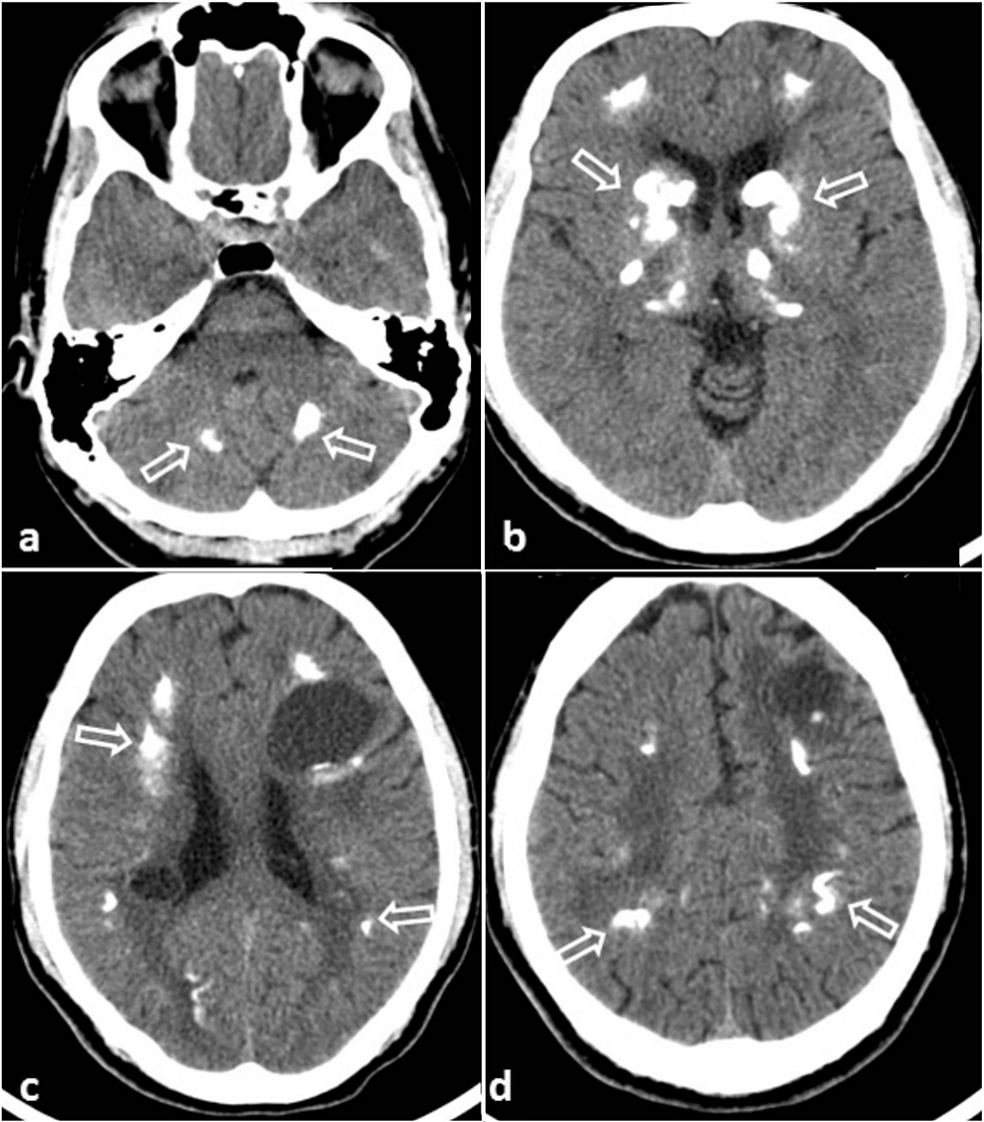
Axial sections of CT brain showing multiple calcific foci in bilateral dentate nuclei (a), basal ganglia (b), and bilateral cerebral white matter (c and d). Also, note the white matter cysts and diffuse white matter hypodensity.

CT scan of the brain revealed symmetric dense calcifications in bilateral basal ganglia, thalami, dentate nuclei, and the white matter of both hemispheres. Calcifications were noted in ventral pons and basal ganglia with diffuse hypodensities in the periventricular white matter. Two cysts were seen in the left frontal and right parietal regions without any surrounding edema and minimal mass effect (Figure 1).

**Figure 2:**
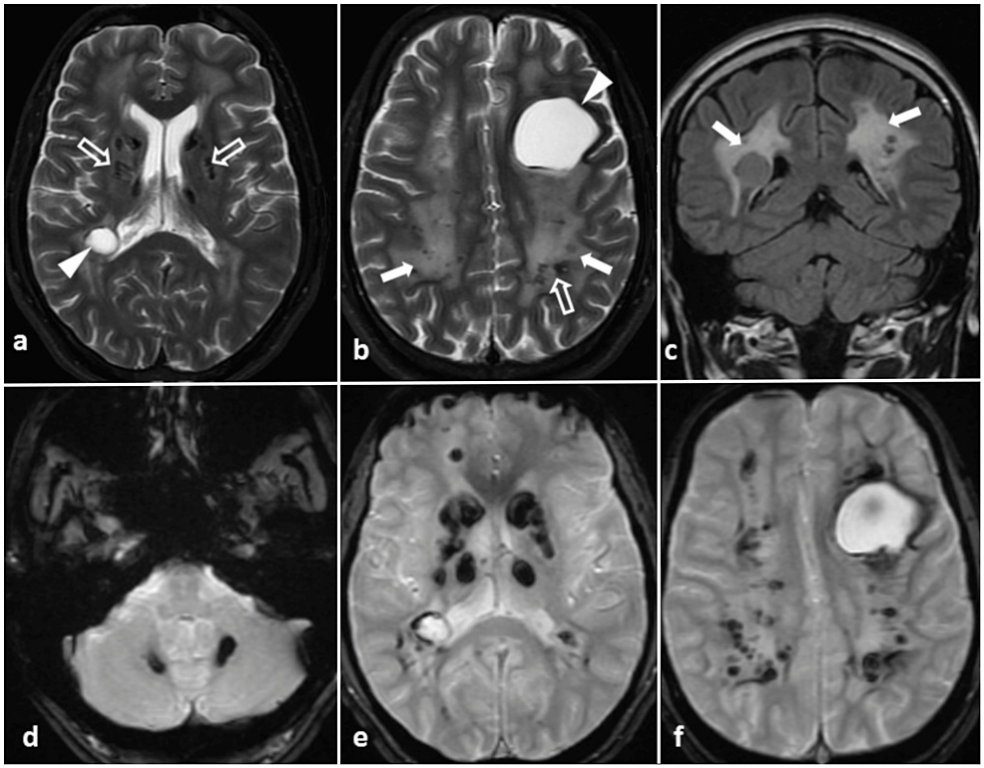
MRI of the brain done at the presentation: axial T2-weighted images (a and b) and coronal T2-FLAIR image (c) show confluent periventricular and deep white matter hyperintensity in both cerebral hemispheres suggestive of leukoencephalopathy (solid arrows) with cystic lesions (arrowheads) and T2 dark foci (hollow arrows). Gradient recalled echo images (d, e, and f) show near symmetric foci of blooming in bilateral dentate nuclei (d), bilateral basal ganglia (e), the white matter of both cerebral hemispheres and along the cyst walls (f). FLAIR: Fluid-attenuated inversion recovery.

Brain MRI revealed confluent areas of T2/Fluid-attenuated inversion recovery (FLAIR) hyperintensities involving the deep periventricular white matter in both cerebral hemispheres with sparing of subcortical U-fibres and two cysts in the left frontal and right posterior temporal region with minimal mass effect and no surrounding edema (Figure 2). On susceptibility-weighted imaging, several foci of blooming in the subcortical white matter with hemosiderin deposition were noted along the cyst walls.

EEG showed an epileptic focus originating from the left frontal region. Seizure control was achieved with levetiracetam, and the patient was placed under close follow-up, with genetic testing offered but not pursued due to financial constraints. The intracranial lesions did not require neurosurgical intervention as they did not cause mass effects. The patient is scheduled for yearly MRIs and six-monthly outpatient visits for follow-up.

## Discussion

The combination of calcifications, cysts, and white matter edema is usually concerning for a parasitic infection, but no serologic evidence was found for the same. Other diseases to be considered include neurocysticercosis (NCC), hydatid disease, and cryptococcosis [6]. Cerebral cysts, basal ganglia calcifications, and supratentorial cysts rarely present together. This case closely corresponds to LCC described in previous studies by Labrune P et al. [1] and Nagae-Poetscher LM et al. [2]. Imaging showed extensive brain calcifications, leukodystrophy, and the formation of parenchymal cysts. When Coats disease presents with leukoencephalopathy, cysts, and calcification, its classified as Coats plus [7]. Coats plus syndrome presents with LCC along with bilateral retinal telangiectasis [7]; however, our patient had a normal ophthalmological evaluation. LCC is characterized by various clinical manifestations such as cognitive decline, convulsive seizures, and pyramidal, extrapyramidal, and cerebellar signs [5]. These clinical features can appear as early as infancy. However, in our case, the patient did not present with any clinical features until he experienced his first generalized seizure at the age of 21. While there remains no consensus on the management, treatment is currently directed at addressing the symptoms, and surgery is offered if the cysts are large enough to cause a mass effect [8]. Further studies are required to elucidate the course of the disease and enunciate the treatment plan. To date, approximately 9-11 case reports have been published, with about 100 reported cases [8,9]. This disease is the first example of a point mutation in a C/D box snoRNA in human disease. Stable RNA-protein complexes, such as SNORD118, with uridine-rich RNA components, are called small nuclear ribonucleoproteins (snRNPs). They are predicted to have a role in pre-mRNA splicing [4]. Being a non-protein-encoding region makes establishing causation difficult [4].

## Conclusions

In conclusion, LCC represents a specific recognizable clinical and radiologic entity, with degenerative cerebral microangiomatous changes being the most prominent histopathologic feature. LCC has a strong genetic predisposition, as highlighted by the clustering of LCC in families. LCC is a genetic disorder caused by mutations in the SNORD118 gene, which produces a type of RNA that is involved in ribosome synthesis but does not encode proteins. It is unclear how this ribosomal dysfunction leads to the specific manifestation of cerebral microangiopathy. To confirm the diagnosis and rule out other potential causes of intracranial cysts with calcifications and leukoencephalopathy, extensive diagnostic testing must be performed before genetic counseling can be provided. Currently, there is no standard protocol for diagnosing or treating LCC, but the disease displays a characteristic imaging pattern, and genetic testing can confirm the SNORD118 gene mutation. Diagnosis can be achieved by excluding similar pathologies, and definitive diagnosis can be achieved through genetic analysis of SNORD118. Treatment is primarily symptomatic, involving surgery to remove mass effects and medical management to control seizures and mental status changes.
